# Multiple Micronutrient Powder Reduces Vitamin E Deficiency in Brazilian Children: A Pragmatic, Controlled Clinical Trial

**DOI:** 10.3390/nu11112730

**Published:** 2019-11-11

**Authors:** Lina M. C. Lobo, Raquel M. Schincaglia, Maria do Rosário G. Peixoto, Maria Claret C. M. Hadler

**Affiliations:** 1 Graduate Program in Health Sciences, Faculty of Medicine, Federal University of Goiás, Goiânia, Goiás 74605-020, Brazil; linamonteiro@gmail.com (L.M.C.L.); raquelms@outlook.com (R.M.S.); 2 Graduate Program in Nutrition and Health, Faculty of Nutrition, Federal University of Goiás, Goiânia, Goiás 74605-080, Brazil; mrg.peixoto@uol.com.br

**Keywords:** vitamin E, tocopherols, health promotion, deficiency diseases, micronutrients, child

## Abstract

Multiple micronutrient powder supplementation is a health promotion strategy, but data on its effectiveness regarding vitamin E are rare. The objective was to evaluate the impact of home fortification with powdered micronutrients on α-tocopherol concentrations, growth, and inflammation in Brazilian children aged 6–15 months. This is a pragmatic, controlled clinical trial, in which the intervention group received micronutrient powder sachets for up to 3 months. Vitamin E deficiency was considered when α-tocopherol was less than 11.6 µmol/L. The Poisson regression model was used to estimate adjusted values for prevalence ratios (PR) for the outcome variable. A total of 224 children participated in the study. The intervention group had a higher median α-tocopherol level (17.2 versus 3.6 µmol/L; *p* < 0.001) and an 82.0% reduction in the prevalence of vitamin deficiency (PR = 0.18; 95% CI 0.11–0.30) when compared with the control group. Consumption of multiple micronutrients in powder increases serum α-tocopherol concentrations, promotes better linear growth, and reduces morbidity in children.

## 1. Introduction

Vitamin E is a fat-soluble antioxidant micronutrient and a group of compounds divided into tocopherols and tocotrienols (α, β, γ, and δ). The form with the highest biological activity is α-tocopherol [1,2]. Vitamin E plays an important role in the protection of polyunsaturated fatty acids and other cell membrane components against free radical oxidation. In addition, it plays an essential role in reproductive and neurological processes by regulating cell proliferation and gene expression [3,4]. The major dietary sources of tocopherols and tocotrienols are vegetable oils, nuts, and seeds [5]. Although vitamin E is a natural component of these foods, low dietary intake of vitamin E sources in preschoolers and schoolchildren has been observed [6,7,8].

International data from studies in both developed and developing countries reveal a highly variable prevalence of vitamin E deficiency (3.6% in Belgium and 71.3% in Tunisia) [9,10], partly due to different cut-offs for vitamin E deficiency used. Results from national surveys showed vitamin E deficiency ranging from 8.8% in Acrelândia—Acre to 82.0% in Goiânia—Goiás [11,12].

Vitamin E deficiency occurs most often in children due to limited vitamin E stores and the intense growth phase. In addition, reduced α-tocopherol concentrations may be caused by diets low in vitamin E in combination with inadequate fat, protein, and energy intake. Low concentrations of vitamin E can cause serious health damage, making children more susceptible to infectious conditions and frequent infections, and it may impair linear growth. In more advanced cases, progressive neurological disorders, spinocerebellar ataxia, and muscle deterioration may occur [1,3,13]. Symptoms associated with vitamin E deficiency may be reversed by supplementation with this micronutrient if it occurs prior to the onset of irreversible damage to the neurological system [14].

In 2011, the World Health Organization (WHO) suggested micronutrient powder supplementation as a strategy for home health promotion. This approach involves the use of powdered sachets containing vitamins and minerals; these can be added to semi-solid foods to be offered to the child [15]. In Brazil, the NutriSus program supplies these sachets in daycare centers linked to the Health at School Program (PSE), and they contain 5 mg tocopherol per sachet, in addition to other micronutrients [16]. The assessment of vitamin E status and the influence of home fortification of vitamins and minerals on this deficiency may help guide public policies to improve vitamin E levels in childhood. 

In recent years, various nutritional strategies for the prevention of nutrient deficiencies have been adopted in Brazil and worldwide, such as the fortification of wheat flour with iron and folic acid [17], nutrient supplementation programs (iron and vitamin A) [18], and nutrition education. Until now, few studies have evaluated the impact of home fortification with multi-micronutrient powder on vitamin E concentrations in children aged 6–15 months [19,20,21,22,23].

The scarcity of data evaluating vitamin E deficiency and the negative repercussions of this deficiency on child health justify this study. Therefore, the aim of this study is to evaluate the impact of home fortification with multiple-micronutrient powder on vitamin E concentrations and subclinical inflammation in children aged 6–15 months in the Central-West region of Brazil.

## 2. Materials and Methods

### 2.1. Study Design and Population

The study is part of a multicenter, pragmatic, controlled clinical trial entitled “Efetividade da fortificação caseira com vitaminas e minerais na prevenção da deficiência de ferro e anemia em crianças menores de um ano: estudo multicêntrico em cidades brasileiras,” developed by the research group of Estudo Nacional de Fortificação Caseira da Alimentação Complementar (ENFAC) in four Brazilian cities (Goiânia, Olinda, Rio Branco, and Porto Alegre), from June 2012 to April 2013. The detailed description of the methodology of this matrix study was previously reported by Cardoso et al. [19]. The sample of the present study consists of children evaluated at the Goiânia Center, Goiás, Brazil. This study was registered at www.ensaiosclinicos.gov.br as RBR-5ktv6b.

The inclusion criteria were age 6–15 months, male or female sex, and use of the primary health care network (Traditional Model) of the city of Goiânia, Goiás. The study sample was divided into two groups: control (age 11–15 months) and intervention (age 6–9 months during the initial phase of the study). Children in the intervention group were evaluated between 4 and 6 months after the start of the intervention at which point they had reached the same age as the control group (11–15 months).

Exclusion criteria included premature infants, twins, reported cases of malaria, Human Immunodeficiency Virus (HIV), tuberculosis or hemoglobinopathies, and those undergoing treatment for anemia at the time of the initial study. All these criteria were considered by the matrix study [19].

### 2.2. Data Collection

The sample size calculation was performed according to the matrix study [19], considering the necessary data for the study of anemia. The increase in mean blood hemoglobin concentration was considered as the main outcome of the matrix study [19]. Considering a test power of 95% and a significance level of 5%, 105 children in each group would be needed to detect a 6 g/L difference between hemoglobin means, with an estimated standard deviation of 12 g/L. The outcome was analyzed according to traditional health care centers [24,25] and the sample predicted for Goiânia according to the age group under study was 210 children. The obtained value was increased by 30% to account for eventual losses and refusals, resulting in a sample of 270 children [19].

**Figure 1:**
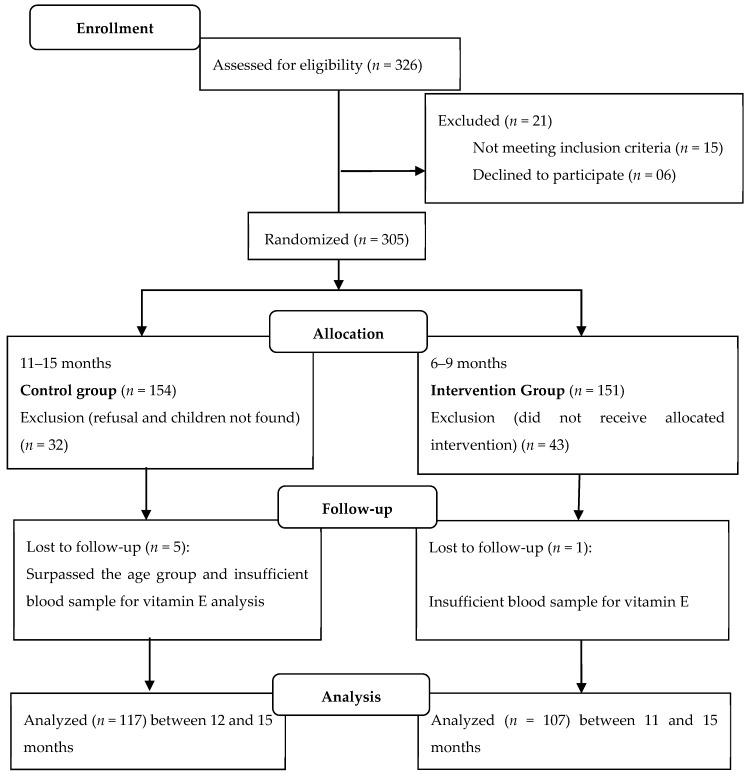
Outline of the matrix project study.

In the present study, 305 children were selected, but blood was not collected from 81 for the following reasons: parental abandonment (*n* = 62), loss of contact with the child (*n* = 13), children who surpassed the proposed age range (*n* = 2), and insufficient blood sample for α-tocopherol evaluation (*n* = 4). The study design is presented in Figure 1.

### 2.3. Groups and Interventions

The control group (CG) was selected at baseline and consisted of children aged 11–15 months. A questionnaire was applied and data collected included socioeconomic, demographic and maternal status, morbidity, food and nutrition of the child, as well as anthropometric measurements of the children and their mothers and venous blood sample collection for hematological and biochemical evaluation of the children.

The intervention group (IG) was selected at the same time as the CG and was composed of children aged 6–9 months attended at the primary health care centers [24,25]. This group was selected to receive micronutrient sachets and be followed for a period of 4–6 months. A similar questionnaire was used in the control group. Children who received the sachets were included in the IG regardless of the degree of adherence to sachet consumption. At this time, the parents and/or guardians answered the same questionnaire applied to the CG on child health, food, and nutrition. Anthropometric measurements were performed on the children and their mothers, and venous blood samples were collected at the end of the study for biochemical evaluation of the children.

The research team advised doctors and nurses on the proper use of sachets and the importance of their use. After this stage, sachets (60 units) containing the formulation of 15 micronutrients were delivered to the parents and/or guardians, and the usage guidelines were given. One sachet was to be offered daily, mixed into semi-solid foods (fruit porridge and salted porridge) at serving time, for a period of 2–3 months. Heating food containing the vitamin and mineral complex was not recommended.

The composition of the sachet followed the criteria adopted by UNICEF (MixMeTM, DSM Nutritional Produts Europe, Ltd., Heerlen, Netherlands) and contained 5 mg of vitamin E, in addition to the following other nutrients: 10 mg iron; 4.1 mg zinc; 150 µg folic acid; 400 µg vitamin A; 30 mg vitamin C; 5 µg vitamin D3; 0.5 mg vitamin B1; 0.5 mg vitamin B2; 0.5 mg vitamin B6; 0.9 µg vitamin B12; 6 mg niacin; 0.56 mg copper; 90 µg iodine; 17 µg selenium.

### 2.4. Anthropometric Assessment

The weight and length of children and the weight and height of their mothers were assessed in two sequential measurements by standardized techniques [26]. For data analysis, the mean value of the two measurements was considered. The weight was measured on a WISO W-835 electronic scale with a 180 kg capacity and 100 g graduations (Zhongshan, Guangdong, China).

Length was determined with a portable Sanny ES-2000 infantometer with a length of 1 m and an accuracy of 1 mm. Children were measured supine on a smooth surface and the value recorded in centimeters. The Z-scores of anthropometric indices (Body Mass Index/age and length/age) were obtained by using the WHO Anthro software version 3.2.2).

### 2.5. Biochemical Assessment

Blood collection was performed at the basic health unit, at home, or in the laboratory by a trained technician experienced in the collection of blood in children. To perform this step, samples of up to 10 mL of venous blood were collected from the children early in the morning after a minimum 3-h fast. The collected blood was distributed into two test tubes, one light-protected serum tube and one plasma EDTA tube.

Serum or plasma was placed in micro-centrifuge tubes and frozen at −20 °C until transport to the Department of Nutrition, Human Nutrition Laboratory (FSP/USP). Samples were stored in the laboratory at −70 °C until biochemical analysis was performed.

The biochemical parameters evaluated in the present study were serum alpha-tocopherol, C-reactive protein (CRP) and alpha-1-glycoprotein (AGP). α-Tocopherol was analyzed by High-Performance Liquid Chromatography (HP-1100 HPLC System, Hewlett-Packard, Palo Alto, CA, USA) [27]. Children with serum α-tocopherol concentrations below 11.6 µmol/L were considered to be vitamin E deficient [28]. For CRP determination, 500 µL of plasma was placed in a clear microtube. CRP and AGP were determined using the IMMAGE Immunochemistry System (Beckman Coulter, Brea, CA, USA). CRP values >5 mg/L and AGP >100 mg/dL were considered as subclinical infection [29,30].

CG children diagnosed with anemia, iron deficiency, or vitamin A deficiency were referred for treatment at the Basic Health Unit, as needed. No IG blood samples were collected at the beginning of the research for ethical reasons, as there would be a likelihood of identifying anemic children without standard iron supplementation treatment.

### 2.6. Statistical Analysis

The database was built using Epi Info, version 5.3.1 for Windows with double entry to check data consistency. Data processing and analysis were performed by using Stata/SE^®^ version 12.0 (StataCorp, College Station, TX, USA).

The continuous independent variables analyzed were age, birth weight, mother’s age, weight, length, length-for-age Z-score (LAZ), number of prenatal consultations, CRP, and AGP. The categorical variables analyzed were gender (female or male), maternal education (<9 years or ≥9 years), current breastfeeding (yes or no), race (white, brown, black), presence of fever (yes or no), cough (yes or no), wheezing (yes or no), and diarrhea (yes or no).

The Shapiro–Wilk test was applied to assess data normality (*p* ≥ 0.05). Sample characterization was performed using absolute and relative frequencies for categorical variables, mean and standard deviation for normally distributed continuous variables, and median and interquartile range for non-normally distributed variables. The Student’s *t*-test or Mann–Whitney test was performed to compare means and medians of independent variables between study groups and Pearson’s chi-square test or Fisher’s exact test to compare observed frequencies between groups. A significance level of 5% was adopted.

Poisson regression analysis of mixed effects with robust variance was used to estimate adjusted values of prevalence ratios (PR) of vitamin E deficiency and inflammation (CRP > 5 mg/L and/or AGP > 100 mg/dL) for the outcome variable. Because of the hierarchical nature of the data, two-level Poisson regression analysis and extra Poisson variation were used to estimate individual-level variance. In the selection of variables by levels, the model maintained those that promoted an increase in the pseudo R^2^ value. The IG was compared with the CG, with adjustment for the variables age of the child, sex, LAZ *Z*-score, race, current breastfeeding, and proximal birth weight; at the distal level, the mother’s age and mother’s education variables were considered. Statistical significance was set at *p* < 0.05, and PR values with 95% CIs were presented.

### 2.7. Ethical Aspects

The study was conducted in compliance with the Helsinki declaration. The study was approved by the Research Ethics Committee of the School of Public Health of the University of São Paulo with protocol n. 2291/2013 and by the Research Ethics Committee of the Federal University of Goiás, with protocol number 065/12. The criteria established by National Health Council Resolution 466/2012, which establishes research standards for humans, were carefully followed.

Data collection was previously authorized by the Municipal Health Department of Goiânia, Goiás. Data were collected from children whose parents or guardians consented to participate in the research and signed the Informed Consent Form (ICF). The matrix study [19] was registered at www.ensaiosclinicos.gov.br with the protocol RBR-5ktv6b.

## 3. Results

A total of 224 children were evaluated, 117 children in the control group and 107 in the intervention group. The characteristics of the children selected for the study are presented in Table 1. By the end of the intervention, 36.8% of the children had consumed 60 sachets (*n* = 39), in compliance with the initial recommendations of the study. These data were estimated according to the number of sachets left.

Although the age variable was not homogeneous between the groups, the mean age difference between the children in the control and intervention groups was 21 days at the end of the study, with a higher mean age for the children in the control group (*p* < 0.001). There was no significant difference between the groups for the variables gender, birth weight, maternal education, mother’s age, current breastfeeding, number of prenatal consultations, and child’s race.

Table 2 presents data on anthropometric, clinical, and biochemical indicators in the study groups. The IG children were longer for age at the end of the study (*p* = 0.02). The proportion of coughing and wheezing episodes was significantly lower in the IG (*p* < 0.001 and *p* = 0.006, respectively). There was a tendency toward a lower prevalence of fever episodes in the IG (*p* = 0.07). The median serum α-tocopherol was significantly higher in the IG (*p* < 0.001). The prevalence of vitamin E deficiency was lower in the IG when compared with the CG (*p* < 0.001). The median CRP and the prevalence of high CRP serum concentration were similar between groups. The median alpha-1-glycoprotein was similar between groups; however, the prevalence of children with high alpha-1-glycoprotein values was lower in the IG (*p* < 0.024).

Analysis of the adjusted prevalence ratio for the variables age of child, sex, *Z*-score for age, race, current breastfeeding, birth weight, mother’s age, and mother’s education showed an 82.0% reduction in prevalence of vitamin E deficiency in the IG when compared with the CG (PR = 0.18; 95% CI 0.11; 0.30; *p* < 0.001). In a similar analysis, a 48% reduction in subclinical inflammation (AGP > 100 mg/dL) was observed in the IG compared with the CG (PR = 0.52; 95% CI 0.31; 0.85; *p* = 0.009). There was no significant difference between groups regarding the prevalence of elevated CRP (Table 3).

## 4. Discussion

Home fortification with multiple powdered micronutrients promoted increased serum tocopherol concentrations in children attending traditional health facilities in Central-Western Brazil and reduced the prevalence of vitamin E deficiency in children in the intervention group compared with those in the control group. It is noteworthy that when compared with the control group, children who received homemade fortification with powdered micronutrients had higher LAZ indices, indicating significant improvement in linear growth, and a lower frequency of cough and wheezing. The final model of this study also showed a significant reduction in the presence of subclinical inflammation in the intervention group, as assessed by AGP.

An important result of the present research was the reduction of vitamin E deficiency in children of the IG when compared with those of the CG. Recent studies have shown a high prevalence of vitamin E deficiency in childhood. According to data from the Estudo Nacional de Fortificação Caseira da Alimentação Complementar (ENFAC), vitamin E insufficiency was found at a prevalence rate of 61.5% in Brazilian children between 6 and 14 months of age [19] and at 82.0% in children of the same age group in Goiânia—Goiás, Brazil [12]. Some factors may contribute to the high prevalence of vitamin E deficiency in this study, such as overall food availability, the vitamin E content of local staple foods, the accessibility and cost of food sources of this nutrient, and the prevalence of infectious conditions.

The effectiveness of fortification with multiple micronutrients, including vitamin E, was also observed in a study by Kumar and Rajagopalan [31], who found a significant increase in mean α-tocopherol in the experimental group after 9 months of intervention in children aged 5–15 years. The authors reported that the increase in this biochemical measurement demonstrated good bioavailability of supplementary micronutrients provided added to food during cooking, including, in this case, good bioavailability of vitamin E. Previous studies have demonstrated good bioavailability of vitamin E in supplemental micronutrients, which might also have influenced the results in our study. It is known that the bioavailability of vitamin E is influenced by the amount of dietary fat and the dietary matrix [32], which may have influenced the serum α-tocopherol concentrations of the children evaluated as the supplement was supplied with the diet.

In studies published by the International Research Group on Infant Supplementation (IRIS), divergent results were observed on the effects of multiple micronutrient supplementation on serum tocopherol concentrations. In one of these studies, Untoro et al. [20] evaluated the effectiveness of multiple micronutrient supplementation in 6- to 12-month-old Indonesian children and found that daily supplementation with chewable tablets for 6 months increased serum α-tocopherol concentrations compared with baseline and in relation to the placebo group, as observed in the multicenter study of this study group [21]. On the other hand, De Romaña et al. [22] performed the same methodology in Peruvian children of the same age and found no association between serum α-tocopherol concentrations and the supply of multiple micronutrients in chewable tablets. It is noteworthy that the serum α-tocopherol concentrations of Peruvian children were lower than the α-tocopherol concentrations of Indian children. The authors concede that this result may have been caused by adequate consumption of this micronutrient in the evaluated children, but this statement contradicts the results of studies that evaluated the dietary intake of vitamin E in childhood [7,8], in which low dietary vitamin intake was found at this stage of life. In addition, other factors should be considered for assessing the impact of supplementation on children, such as health conditions and the inflammatory profile of the patient, as this state of health may be a detrimental factor for the absorption of several nutrients, including vitamin E. Thus, the high levels of infection in the evaluated patients may have been high enough to inhibit the use of vitamin E in the supplementation offered.

The importance of home fortification in aiding in children’s growth was evidenced by the results of this study. However, few children in this study had impaired linear growth. As the LAZ index indicates the cumulative effect of adverse situations on children’s growth and is a sensitive indicator for measuring the quality of life of the population [33], nutritional strategies that promote higher linear growth in childhood, such as home fortification with powdered micronutrients, become essential for supporting proper growth of the child.

The duration of the intervention (2–3 months) and the α-tocopherol dose of 5 mg promoted an increase in vitamin E levels in the children evaluated. This dose corresponds to AI values for children aged six to 12 months, which is 5 mg/day, and for children aged one to three years (6 mg/day). The DRIs also establish an estimated average requirement (EAR) of 5 mg/day vitamin E for children aged one to three years [34]. However, the offer of micronutrient powder sachets containing vitamin E did not fully reduce the deficiency of this nutrient among the children studied. A similar time of treatment and a similar dose were used by other authors [20,21,23]. 

Nutritional deficiencies may occur simultaneously in childhood [20]. Thus, nutritional interventions with unique nutrients may not be sufficient in places where there are multiple nutrient deficiencies [21]. Previous studies have shown that multiple nutrient administration can improve linear growth in children [23,35]. The occurrence of a higher rate of linear growth in the intervention group of the present study relative to the control group may be explained by the following factors: the frequency of diarrhea was similar between the groups but episodes of fever and wheezing in the 15 days prior to the survey were lower in the IG than in the CG at the end of the study. These results may be related to better childhood health conditions that support adequate growth.

Point-of-use fortification at home with multiple powdered micronutrients promoted a lower prevalence of some morbidity indicators, such as coughing and wheezing, and tended to lower the prevalence of fever in the intervention group, but there was no difference in the frequency of diarrhea episodes between the evaluated groups. These data corroborate the findings of De Romaña et al. [22], in which the authors report that the improvement in respiratory symptoms may reflect seasonal variations and that the lack of association between supplementation and fever and diarrhea is difficult to explain, since prophylactic nutrient supplementation may assist in reducing childhood morbidity, and the amount of nutrients provided in supplementation may have been below what was necessary to verify this effect. It is noteworthy that the antioxidant role and the improvement of the immune response caused by vitamin E consumption [36] may have contributed to the improved morbidity indicators in this study. The effectiveness of supplementation in improving nutrient status and growth depends on intestinal health for the absorption and inflammatory state of the child [37]. In our study, the fact that so few have impaired linear growth indicates that living conditions for these children were not growth restraining. The presence of inflammation was higher in the control group than in the intervention group, with a significant difference in subclinical inflammation determined by AGP. These data suggest that there was a better health profile in the intervention group at the end of the study. Suchdev et al. [38] report that the prevalence of high AGP is substantially higher than the prevalence of high CRP, so AGP seems to have a major impact on nutritional biomarkers at the population level.

To our knowledge, this is the first study from the Central-West region of Brazil to evaluate the effectiveness of home fortification of vitamins and minerals on vitamin E concentrations in children treated at health facilities and provides important results to strengthen public health policies aimed at reducing this nutritional deficiency in childhood. The ENFAC study evaluated the effectiveness of multi-nutrient home fortification in four Brazilian regions, but the data were not analyzed separately at each center. On the other hand, the methodological design of our study did not allow blinding, thus constituting a limitation. Potential sample biases were reduced by blindly performing laboratory procedures during data collection, and the two groups were compared when both were the same age. The anthropometric evaluation performed only on a single occasion represents a limitation of this study [39] because home fortification performed during the age of rapid growth may have influenced the results of the intervention group and the design of the study did not permit the assessment of anthropometric monitoring from the beginning to the end of the study. However, despite these aspects, it was possible to detect a significant difference in Z-score of LAZ between the control and intervention groups when both reached the same age at the end of the study. The influence of seasons of the year on the indicators of morbidity was not evaluated, representing a limitation of the study. Importantly to highlight the difficulty of evaluating the data from the present study due to the scarcity of studies on the effectiveness of home fortification on vitamin E concentrations in children under 14 months of age; for this reason, other studies that evaluated children over 2 years of age were used.

## 5. Conclusions

At home fortification with multiple powdered micronutrients provided once daily for a period of 2–3 months reduces the prevalence of vitamin E deficiency, increases serum tocopherol concentrations, and promotes better linear growth and morbidity profiles in 4 to 6 months after the start of fortification. Subclinical inflammation was lower in the intervention group, suggesting a better health profile among children who received homemade fortification with multiple powdered micronutrients. Food intervention through fortification with powdered micronutrients as part of the routine of Traditional Health Units from 6 to 8 months of age may be a strategy to improve the vitamin E profile and reduce the high prevalence of this deficiency in childhood.

## Figures and Tables

**Table 1:** Characteristics of children participating in the ENFAC study (Goiânia, 2013; *n* = 224).

Variable	CG (*n* = 117)	IG (*n* = 107)	*p* Value
Age of child (months), mean, and SD	13.65 ± 0.87	12.94 ± 0.94	<0.001 ^1^
Female gender, *n* (%)	55 (47.01)	53 (49.53)	0.71 ^2^
Birth weight, mean and SD	3247.5 ± 469.8	3288.5 ± 426.1	0.49 ^3^
Maternal education <9 years, *n* (%)	33 (28.45)	21 (19.81)	0.13 ^2^
Mother’s age (years), median (IQR)	28 (24; 32)	27 (22; 31)	0.18 ^1^
Current breastfeeding, *n* (%)	67 (57.26)	59 (55.14)	0.75 ^2^
Number of prenatal consultations, median (IQR)	8 (6;10)	8 (6;9)	0.80 ^1^
Race, *n* (%)			0.95 ^4^
White	9 (7.76)	10 (9.35)	
Brown	102 (87.93)	93 (86.92)	
Black	5 (4.31)	4 (3.74)	

^1^ Mann–Whitney; ^2^ Pearson’s chi-square test; ^3^ Student’s *t*-test; ^4^ Fisher’s exact test. CG: control group; ENFAC: Estudo Nacional de Fortificação Caseira da Alimentação Complementar; IG: intervention group; IQR: interquartile range; SD: standard deviation.

**Table 2:** Anthropometric, clinical, and biochemical data on participating children at the end of the ENFAC study (Goiânia, 2013; *n* = 224).

Variable	CG (*n* = 117)	IG (*n* = 107)	*p* Value
**Anthropometric data**			
Weight (kg), mean and SD	10.02 ± 1.20	9.85 ± 1.19	0.29 ^1^
Length (cm), median (IQR)	77 (75; 79)	77.8 (75; 79.5)	0.68 ^2^
LAZ (*Z*-score), mean and SD	0.07 ± 1.13	0.41 ± 1.09	0.02 ^1^
**Clinical data**			
Presence of fever, *n* (%)	39 (33.33)	24 (22.43)	0.07 ^3^
Presence of cough, *n* (%)	53 (45.30)	21 (19.63)	<0.001 ^3^
Presence of wheezing, *n* (%)	35 (29.91)	16 (14.95)	0.008 ^3^
Presence of diarrhea, *n* (%)	27 (23.08)	22 (20.56)	0.65 ^3^
**Biochemical data**			
Serum α-tocopherol (µmol/L), median (IQR)	3.6 (0.09; 9.7)	17.2 (12.9; 21.2)	<0.001 ^2^
CRP (mg/L), median (IQR)	0.5 (0.2; 1.5)	0.3 (0.2; 1.0)	0.19 ^2^
AGP (mg/dL), median (IQR)	74.5 (61.8; 109.5)	72.4 (61.4; 92.3)	0.08 ^2^

^1^ Student’s *t*-test; ^2^ Mann–Whitney test; ^3^ Pearson’s chi-square. AGP: alpha-1-glycoprotein; CG: control group; CRP: C-reactive protein; ENFAC: Estudo Nacional de Fortificação Caseira da Alimentação Complementar; IG: intervention group; IQR: interquartile range; LAZ: length-for-age Z-score; SD: standard deviation.

**Table 3:** Prevalence and prevalence ratio for vitamin E deficiency and inflammatory status between control and intervention groups at the end of the ENFAC study (Goiânia, 2013; *n* = 224).

Variable	CG (*n* = 117)	IG (*n* = 107)	*p* Value
**Biochemical data**			
Vitamin E deficiency (<11.6 µmol/L), *n* (%)	95 (85.6)	16 (14.4)	<0.001 ^1^
Vitamin E deficiency, adjusted PR (95% CI)	1	0.18 (0.11; 0.30)	<0.001 ^2^
CRP > 5 mg/L, *n* (%)	14 (12.39)	6 (5.83)	0.096 ^1^
CRP > 5 mg/L, adjusted PR (95% CI)	1	0.40 (0.13; 1.29)	0.13 ^2^
AGP > 100 mg/dL, *n* (%)	37 (33.04)	20 (19.42)	0.024 ^1^
AGP, adjusted PR (95% CI)	1	0.52 (0.31; 0.85)	0.009 ^3^

^1^ Pearson’s chi-square; ^2^ Null hypothesis test between groups using the mixed-effect Poisson regression model adjusted for the child’s age, gender, length-for-age Z-score, race, current breastfeeding, birth weight, mother’s age, and mother’s schooling. AGP: alpha-1-glycoprotein; CG, control group; CRP, C-reactive protein; IG, intervention group; PR: prevalence ratio.

## Abbreviations

AGP	Alpha-1-glycoprotein
CG	Control group
CRP	C-reactive protein
ENFAC	Estudo Nacional de Fortificação Caseira da Alimentação Complementar
ICF	Informed Consent Form
HIV	Human Immunodeficiency Virus
HPLC	High Performance Liquid Chromatography
IG	Intervention group
IRIS	International Research on Infant Supplementation
IQR	Interquartile range
PSE	Health at School Program
PR	Prevalence ratio
SD	Standard deviation
WHO	World Health Organization
